# CNS tumors with *YWHAE:NUTM2* and *KDM2B*-fusions present molecular similarities to extra-CNS tumors having *BCOR* internal tandem duplication or alternative fusions

**DOI:** 10.1186/s40478-021-01279-3

**Published:** 2021-10-30

**Authors:** Arnault Tauziède-Espariat, Gaëlle Pierron, Delphine Guillemot, Dorian Bochaton, Sarah Watson, Julien Masliah-Planchon, Alexandre Vasiljevic, Alexandra Meurgey, Guillaume Chotard, Lauren Hasty, Ellen Wahler, Emmanuèle Lechapt, Fabrice Chrétien, Jacques Grill, Franck Bourdeaut, Yassine Bouchoucha, Stéphanie Puget, Céline Icher-de-Bouyn, Vincent Jecko, Liesbeth Cardoen, Volodia Dangouloff-Ros, Nathalie Boddaert, Pascale Varlet, on behalf of the RENOCLIP-LOC

**Affiliations:** 1 Department of Neuropathology, GHU Paris - Psychiatry and Neuroscience, Sainte-Anne Hospital, 1 Rue Cabanis, 75014 Paris, France; 2 Paris-Sciences-Lettres, Curie Institute Research Center, INSERMU830, Paris, France; 3 Laboratory of Somatic Genetics, Curie Institute Hospital, Paris, France; 4 Department of Medical Oncology, Curie Institute Hospital, Paris, France; 5 Department of Pathology and Neuropathology, GHE, Hospices Civils de Lyon, Lyon, France; 6 Department of Biopathology, Léon Bérard Cancer Center, Lyon, France; 7 Department of Pathology, Groupe Hospitalier Pellegrin, CHU de Bordeaux, Bordeaux, France; 8 Department of Oncology for Child and Adolescents, Gustave Roussy, Villejuif, France; 9 SIREDO Center Care, Innovation, Research In Pediatric, Adolescent and Young Adult Oncology, Curie Institute and Paris Descartes University, Paris, France; 10 Université de Paris, Paris, France; 11 Department of Pediatric Neurosurgery, Hôpital Universitaire Necker Enfants Malades, APHP, Université de Paris, Paris, France; 12 Department of Pediatrics, Bordeaux University Hospital, 33076 Bordeaux, France; 13 Department of Neurosurgery A Unit, Bordeaux University Hospital, 33076 Bordeaux, France; 14 Department of Radiology, Curie Institute, Paris University, 75005 Paris, France; 15 Pediatrics Radiology Department, Hôpital Necker Enfants Malades, AP-HP, University de Paris, INSERM U1163, Institut Imagine, Paris, France

*BCOR* (*BCL6 Corepressor*) internal tandem duplication (ITD) has been implicated in a wide variety of tumors of different organs [1]: clear cell sarcomas of the kidney (CCSK), high-grade endometrial stromal sarcomas (HGESS), undifferentiated round cell sarcomas (URCS) in the soft tissue, and tumors within the central nervous system (CNS). BCOR is part of the polycomb repressive complex 1.1 (PRC1.1), in association with the KDM2B (Lysine Demethylase 2B) protein, that mediates transcriptional repression of oncosuppressors through post-translational modifications of histones. A variable proportion of CCSK, HGESS and URCS present the *YWHAE:NUTM2* fusion which is always found in mutual exclusion with the *BCOR* ITD. Tumors with *YWHAE:NUTM2* fusions also exhibit *BCOR* up-regulation, reinforcing the hypothesis that these two alterations activate a common pathogenetic pathway [2, 3]. CNS tumors, isolated from a series of primitive neuroectodermal tumors by a distinct methylation profile, were initially named high-grade neuroepithelial tumors (HGNET) with *BCOR* alteration [4]. Because almost all HGNET-*BCOR* harbored *BCOR* ITD and of an unknown cellular origin, the cIMPACT-NOW update 6 recommends the terminology “CNS tumor with *BCOR* ITD” [4–6]. Here, we report four CNS tumors with *YWHAE:NUTM2* or *KDM2B* fusions, which did not cluster with HGNET-*BCOR* by DNA-methylation analysis, but clustered with extra-CNS sarcomas with *BCOR* ITD or analog fusions.

These pediatric cases included a 1-year old boy (Case 1), a 7-year old boy (Case 2), a 10-year old girl (Case 3), and a 7-year old girl (Case 4). Tumors were located in the left temporal dura-mater (Case 1), the left temporal and parietal lobes (Case 2), the right parietal and occipital lobes (Case 3), and in the pons (Case 4). Case 1 was an extra-axial mass with an intense and homogeneous enhancement, and a restricted apparent diffusion coefficient (ADC), reflecting high cellularity (Fig. 1A). Case 2 was a large and well-circumscribed solid tumor with hemorrhaging and necrosis, and a slight enhancement after contrast injection (Fig. 1E). Case 3 was a well-circumscribed polycystic tumor with an intense enhancement after contrast injection and slight hypersignal on diffusion weighted images (Fig. 1I). Case #4 was a multinodular enhancing mass in the pons with non-enhancing additional tumoral infiltration on FLAIR sequence (Fig. 1M). Whole body imaging did not evidence a spinal, leptomeningeal or extra-cranial location. Three patients were alive at the end of the follow-up (12, 11, and 179 months, respectively for Cases 1, 2, and 3), only Case 4 died postoperatively. Histopathologically, these tumors were circumscribed from the parenchyma. There was an intra-tumoral hetereogeneity: oligodendroglial-like, undifferentiated (Fig. 1B and N), or ependymal features with pseudorosettes (Fig. 1F and J). Microcysts containing a myxoid substance and calcifications were respectively observed in Cases 2 and 3. Features of malignancy were obvious with necrosis, a high mitotic count and proliferation index, and microvascular proliferation in both cases. Using immunohistochemistry, there was a preserved expression of INI1 and BRG1 and no immunoexpression of LIN28A. The expression of GFAP and Olig2 was absent in three cases and focal in the last tumor. Expression of at least one neuronal marker (NeuN and neurofilaments) was present in three cases. A BCOR immunoexpression was absent or only focally present in 3/4 cases (Fig. 1D, H and L). Analysis of the RNA-seq data identified *YWHAE:NUTM2A* (Case 1), *KDM2B:NUTM2B* (Case 2), *YAP1:KDM2B* (Case 3), and *CHST11:KDM2B (*Case 4) fusions and confirmed by two of the five different methods of detection we use (Defuse V0.6.2, StarFusion v1.2.0 (STAR v 2.5.4a), Fusion Catcher v1.00, FusionMap (Oshell toolkit v10.0.1.50) and ARRIBA v1.2.0) (Fig. 2). Using the Heidelberg DNA methylation classifier, Cases 1 and 4 were not classified and Cases 2 and 3 were classified as HGNET-*BCOR* (with calibrated max-scores of 0.8 and 0.2). To better characterize the potential cellular origin of these cases, we performed a t-Distributed Stochastic Neighbor Embedding plot (t-SNE) analysis including HGNET-*BCOR* and sarcomas with *BCOR* alterations (HGESS and URCS, also referred to as Small Blue Round Cell Tumours—SBRCT—in the Heidelberg database) and four soft tissue sarcomas with *YWHAE:NUTM2* or *KDM2B* fusions from our in-house database (Fig. 3). Interestingly, our four CNS cases clustered in close vicinity with sarcomas and not HGNET-*BCOR* (Fig. 3).

**Fig. 1:**
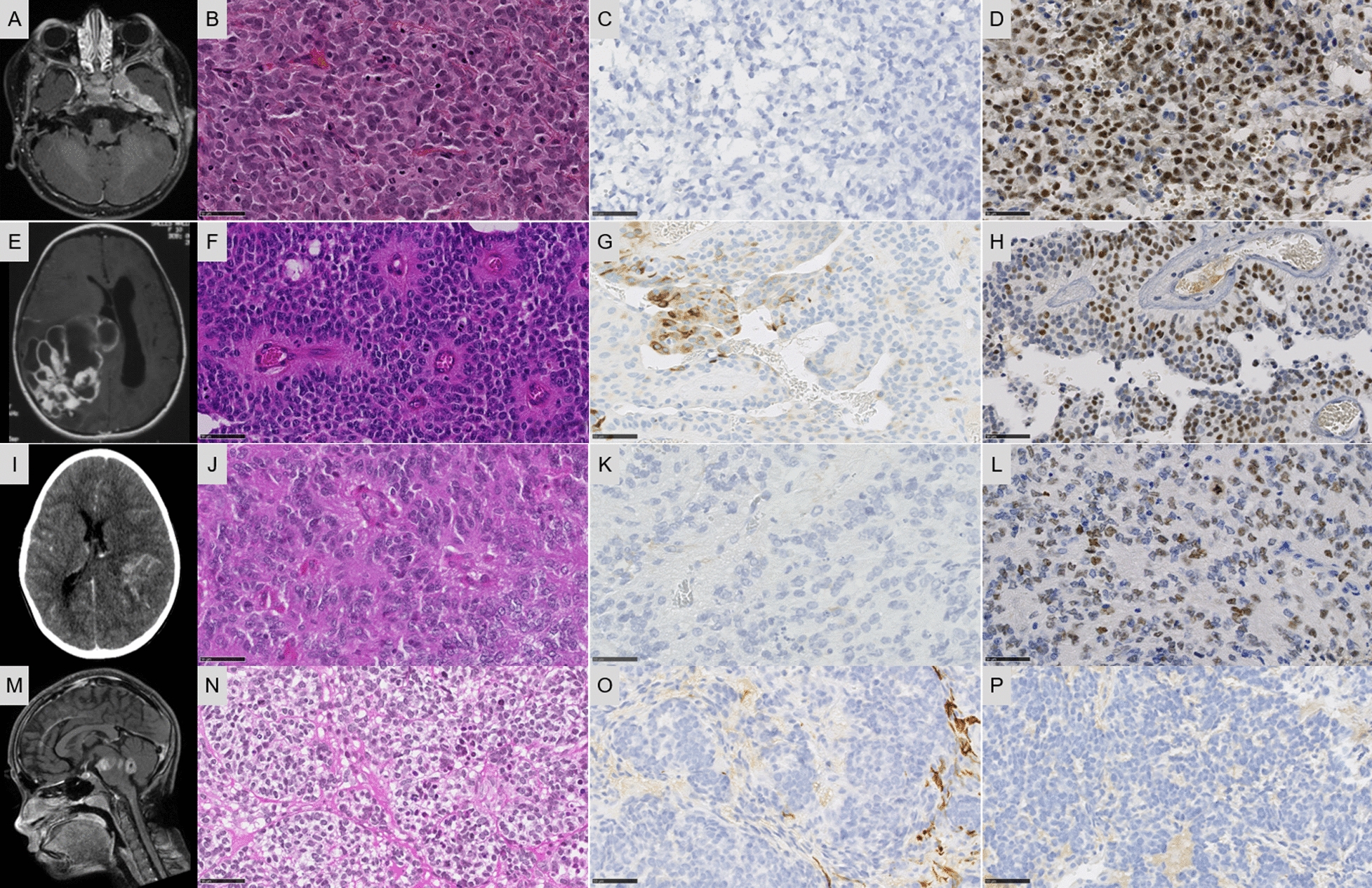
Radiological and histopathological features. **A** Axial T1-weighted sequence with gadolinium showing an enhanced extra-axial left temporal mass. **B** Compact tumor with undifferentiated morphology (HPS, magnification × 400). **C** No immunoexpression of GFAP (magnification × 400). **D** Expression of BCOR in tumor cells (magnification × 400). **E** Axial T1-weighted sequence with gadolinium showing an enhancing polycystic mass in the right parietal lobe. **F** Proliferation composed of pseudorosettes (HPS, magnification × 400). **G** Focal expression of GFAP (magnification × 400). **H** BCOR immunoexpression in a part of tumor cells (magnification × 400). **I** Axial tomodensitometry with contrast showing a hemorrhagic lesion of the left parietal lobe with slight contrast enhancement. **J** Proliferation composed of pseudorosettes (HPS, magnification × 400). **K** Absence of immunoexpression of GFAP (magnification × 400). **L** Expression of BCOR (magnification × 400). **M** Sagittal T1-weighted sequence with gadolinium showing an enhancing multinodular lesion of the pons. **N** Compact tumor with nodular arrangement of undifferentiated cells (HPS, magnification × 400). **O** No immunoexpression of GFAP (magnification × 400). **P** No immunoexpression of BCOR (magnification × 400). Black scale bars represent 50 µm. Each line represents a case: Case 1 for A-D pictures; Case 2 for E–H pictures, Case 3 for I-L pictures, and Case 4 for M-P pictures. HPS: Hematoxylin Phloxin Saffron

**Fig. 2:**
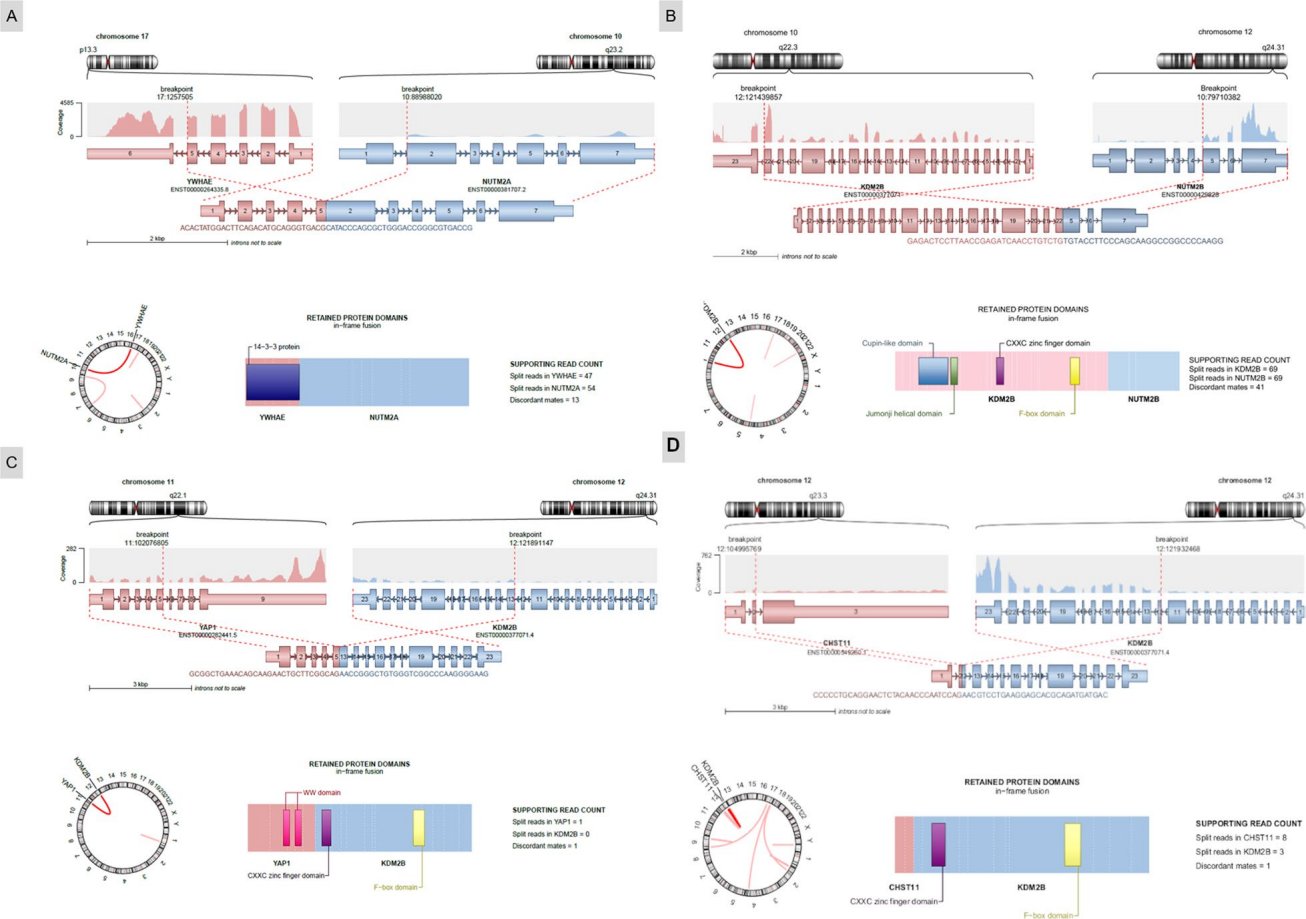
Genetic features. **A** RNAseq analysis highlights a fusion between *YWHAE* (pink) and *NUTM2A* (blue) genes, respectively located on chr17p13.3 and chr10q23.2. As the breakpoints are intra exonic (in exon 5 for *YWHAE*, and exon 2 for *NUTM2A*), the fusion point can easily been detected by split and span reads encompassing the rearrangement with a good coverage. Localized on minus strand (inverse orientation), the DNA sequence of *NUTM2A* is switched in frame with *YWHAE* (Circos plot). **B** RNAseq analysis highlights a fusion between *KDM2B* (pink) and *NUTM2B* (blue) genes, respectively located on chr10.q22.3 and chr12.q24.31. As the breakpoints are intra exonic (in exon 22 for *KDM2B*, and exon 5 for *NUTM2B*), the fusion point can easily been detected by split and span reads encompassing the rearrangement with a good coverage. Localized on minus strand (inverse orientation), the DNA sequence of *NUTM2B* is switched in frame with *KDM2B* (Circos plot). **C** RNAseq analysis highlights a fusion between *YAP1* (pink) and *KDM2B* (blue) genes, respectively located on chr11q22.1 and chr12q24.31. As the breakpoints are intra exonic (in exon 5 for *YAP1*, and exon 13 for *KDM2B*), the fusion point can easily been detected by split and span reads encompassing the rearrangement with a good coverage. Localized on minus strand (inverse orientation), the DNA sequence of *KDM2B* is switched in frame with *YAP1* (Circos plot). For this case, the quality of the method of detection was not perfect and we cannot affirm that it was not a reciprocal fusion. **D** RNAseq analysis highlights a fusion between *CHST11* (pink) and *KDM2B* (blue) genes, respectively located on chr12q23.3 and chr12q24.31. As the breakpoints are intra exonic (in exon 2 for *CHST11*, and exon 12 for *KDM2B*), the fusion point can easily been detected by split and span reads encompassing the rearrangement with a good coverage. Localized on minus strand (inverse orientation), the DNA sequence of *KDM2B* is switched in frame with *CHST11* (Circos plot). The representations of fusion transcripts were obtained by RNA-seq analysis and built by the ARRIBA bioinformatics fusion finder tool which sometimes highlights reciprocal fusions. The fusion transcripts between the 5' gene (pink) and the 3' gene (blue) are represented on the left. The sequences of the fusion points are written below the transcript pattern. The fusions are systematically exon-exon and preserve the reading frame. The right columns show the fusion proteins and the domains retained by the fusions. Protein domains legend: 14-3-3: 14-3-3 domain superfamily; JmjC: Jumonji family of transcription factors; Cxxc: Zinc finger CXXC-type; Fbox: F-box-like domain superfamily

**Fig. 3:**
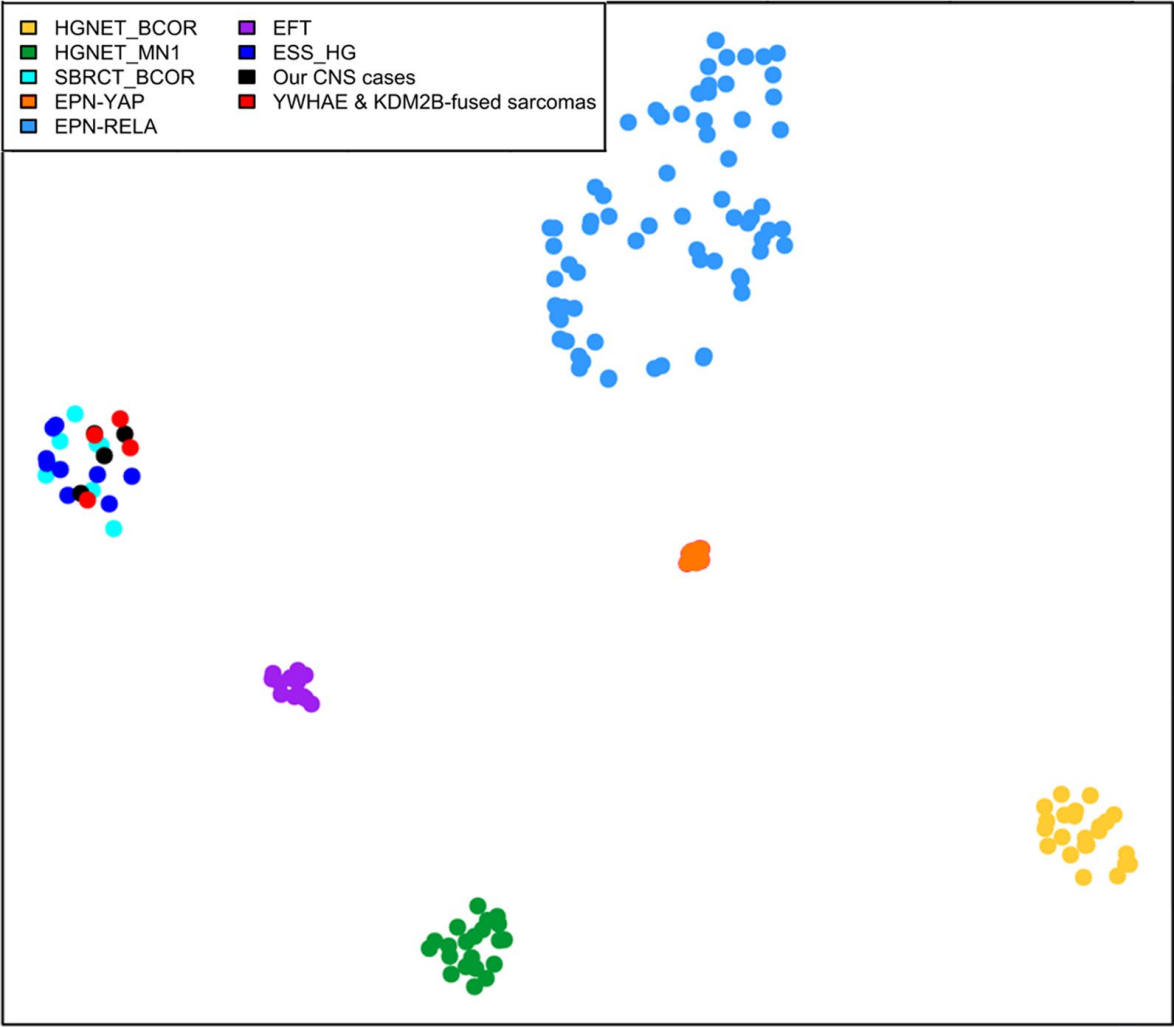
t-SNE distributions based on DNA Methylation and RNA-sequencing. The t-SNE is built with the 10 first principal components (PC) of the DNA methylation $\beta$-values with a standard deviation superior to 20%. Our four tumors were compared with 154 reference samples from the Heidelberg CNS tumors and sarcomas cohorts, belonging to the HGNET-*BCOR* (n = 23)*,* HGNET-*MN1* (n = 21), EPN-*RELA* (n = 70), EPN-*YAP* (n = 10), EFT-CIC (n = 13), SBRCT-*BCOR* (n = 8) and ESS HG (n = 9) methylation classes. We added four in-house sarcomas with *YWHAE:NUTM2* or *KDM2B* fusions. Our four tumors clustered with sarcomas and not HGNET-*BCOR*. HGNET-*BCOR*: high-grade neuroepithelial tumors with *BCOR* alteration; HGNET-*MN1*, high-grade neuroepithelial tumors with *MN1* alteration; EPN-*RELA*, ependymomas with *RELA* fusion; EPN-*YAP*, ependymomas with *YAP* fusion; EFT-*CIC*, Ewing’s sarcoma family of tumors (with *CIC* alteration); SBRCT-*BCOR*, small blue round cell tumors with *BCOR* alteration; ESS HG, high grade endometrial stromal sarcomas

Contrary to HGNET-*BCOR*, CCSK and HGESS may present either *BCOR* ITD or *YWHAE:NUTM2A/B* fusions [3]. Here, we report four CNS tumors harboring *YWHAE:NUTM2* or *KDM2B* fusions which differed from classical CNS tumors with *BCOR* ITD by clinical, radiological, immunophenotypical and molecular findings. Indeed, whereas the prognosis of HGNET-*BCOR* is poor [5], the outcome of our cases seems to be better, except for Case 4 most likely due to the tumoral pontine location. Imaging of our cases was different from those described in HGNET-*BCOR* such as large, solid and well-circumscribed intra-axial tumors that abut the overlying dura, with restricted diffusion and weak heterogeneous enhancement after contrast injection [6, 7]. Morphologically, all tumors were undifferentiated or arranged in small nodules of round clear cells, mimicking CCSK [7]. However, two cases presented ependymoma-like features with pseudorosettes, which have never been seen in extra-CNS tumors, contrary to CNS tumors with *BCOR* ITD. Immunohistochemically, only 1/4 of these tumors expressed Olig2, which is classically diffuse in CNS tumors with *BCOR* ITD [6, 7]. As previously described, BCOR immunoexpression was absent or focal in our cases without *BCOR* ITD [8, 9]. Lastly, DNA methylation clustering showed a close proximity of these cases to sarcomas with *BCOR* alterations. Because DNA methylation profiles are thought to represent a combination of both somatically acquired DNA methylation changes and a signature reflecting the cell of origin, and because no extra-CNS lesion was found in our cases, it is therefore reasonable to believe that they represent another tumor type than classical CNS tumors with *BCOR* ITD. Here, we report for the first time fusions implicating the *KDM2B* gene in the CNS; interestingly, only one case was previously described with a *EPC1-KDM2B* fusion in soft tissue [10]. *KDM2D* fusions were also found in HGESS and SBRCT [11].

To conclude, *YWHAE:NUTM2* and *KDM2B*-fused CNS tumors aggregate within the category of sarcomas with *BCOR* alterations based on their DNA methylation signatures, despite some morphological features mimicking CNS tumors with *BCOR* ITD. Our results suggest that CNS tumors with these types of fusions represent a CNS location of mesenchymal tumors, but more cases (with DNA methylation and expression analyses) are needed for confirmation. Current diagnostic tools involving automated classification based on DNA methylation were proved inaccurate in two of our four cases. The diagnosis of CNS tumors, *BCOR* ITD must therefore be ascertained by precise clustering studies.

## References

[1] Astolfi A, Fiore M, Melchionda F, Indio V, Bertuccio SN, Pession A (2019). BCOR involvement in cancer. Epigenomics.

[2] Kenny C, Bausenwein S, Lazaro A, Furtwängler R, Gooskens SLM, van den Heuvel EM (2016). Mutually exclusive BCOR internal tandem duplications and YWHAE-NUTM2 fusions in clear cell sarcoma of kidney: not the full story. J Pathol.

[3] Momeni-Boroujeni A, Mohammad N, Wolber R, Yip S, Köbel M, Dickson BC (2020). Targeted RNA expression profiling identifies high-grade endometrial stromal sarcoma as a clinically relevant molecular subtype of uterine sarcoma. Mod Pathol.

[4] Sturm D, Orr BA, Toprak UH, Hovestadt V, Jones DTW, Capper D (2016). New brain tumor entities emerge from molecular classification of CNS-PNETs. Cell.

[5] Appay R, Macagno N, Padovani L, Korshunov A, Kool M, André N (2017). HGNET-BCOR tumors of the cerebellum: clinicopathologic and molecular characterization of 3 cases. Am J Surg Pathol.

[6] Ferris SP, Velazquez Vega J, Aboian M, Lee JC, Van Ziffle J, Onodera C (2019). High-grade neuroepithelial tumor with BCOR exon 15 internal tandem duplication-a comprehensive clinical, radiographic, pathologic, and genomic analysis. Brain Pathol.

[7] Yoshida Y, Nobusawa S, Nakata S, Nakada M, Arakawa Y, Mineharu Y (2018). CNS high-grade neuroepithelial tumor with BCOR internal tandem duplication: a comparison with its counterparts in the kidney and soft tissue. Brain Pathol.

[8] Mardi L, Tauziède-Espariat A, Guillemot D, Pierron G, Gigant P, Mehdi L (2021). Bcor immunohistochemistry, and not SATB2, is a sensitive and specific diagnostic biomarker for Cns tumors with BCOR internal tandem duplication. Histopathology..

[9] Chiang S, Lee C-H, Stewart CJR, Oliva E, Hoang LN, Ali RH (2017). BCOR is a robust diagnostic immunohistochemical marker of genetically diverse high-grade endometrial stromal sarcoma, including tumors exhibiting variant morphology. Mod Pathol.

[10] Teramura Y, Tanaka M, Yamazaki Y, Yamashita K, Takazawa Y, Ae K (2020). Identification of Novel Fusion Genes in Bone and Soft Tissue Sarcoma and Their Implication in the Generation of a Mouse Model. Cancers.

[11] Lin DI, Hemmerich A, Edgerly C, Duncan D, Severson EA, Huang RSP (2020). Genomic profiling of BCOR-rearranged uterine sarcomas reveals novel gene fusion partners, frequent CDK4 amplification and CDKN2A loss. Gynecol Oncol.

